# Age‐ and endometrial microbiota‐related delay in development of endometrial receptivity

**DOI:** 10.1002/rmb2.12523

**Published:** 2023-06-27

**Authors:** Shunsaku Fujii, Takaaki Oguchi

**Affiliations:** ^1^ ef.clinic Aomori Japan

**Keywords:** aging, assisted reproductive techniques, endometrium, infertility, microbiota

## Abstract

**Purpose:**

We evaluated factors affecting the development of endometrial receptivity according to age and changes in the endometrial microbiota.

**Methods:**

We recruited patients with infertility who underwent transcriptomic analyses of endometrial receptivity and the endometrial microbiome prior to frozen embryo transfer. An endometrial biopsy was performed 108 h after initial progesterone administration.

**Results:**

In 185 tests from 185 eligible patients, the results of endometrial receptivity analysis were receptive in 111 (60.0%) patients and pre‐receptive in 74 (40.0%) patients. Compared with receptive patients, pre‐receptive patients had significantly older ages (36.0 ± 0.5 vs. 38.2 ± 0.5, *p* = 0.0021), a smaller proportion of normal *Lactobacillus*‐dominant microbiota (27.9% vs. 12.2%), and a greater proportion of microbiota with ultralow biomass (22.5% vs. 41.9%) (*p* = 0.0074). Patient age (adjusted odds ratio: 1.08, 95% confidence interval: 1.01–1.16, *p* = 0.0351) and a microbiome with ultralow biomass (adjusted odds ratio: 3.82, 95% confidence interval: 1.49–9.82, *p* = 0.0039) were independent predictive factors for pre‐receptive endometrium.

**Conclusions:**

Older age was accompanied by a decrease in *Lactobacillus*‐dominant microbiota; aging and endometrial microbiota with ultralow biomass were significantly associated with pre‐receptive endometrium. Our findings suggest that the quantity (rather than proportion) of *Lactobacillus* in the endometrium is important in the development of endometrial receptivity.

## Abstract 2

Older age was accompanied by a decrease in Lactobacillus‐dominant microbiota, and aging and endometrial microbiota with ultralow biomass were significantly associated with pre‐receptive endometrium, suggesting that the quantity rather than the proportion of Lactobacillus in the endometrium might be important in the developmental process of endometrial receptivity.

## 1 INTRODUCTION

Endometrial receptivity for embryos is an important but minimally investigated topic in assisted reproductive technology (ART). With advancements in genome technology, the endometrial receptivity array has become established as a novel diagnostic tool for measuring potential transcriptomic profiles related to embryo implantation;^1^ this approach is more accurate and consistent than conventional analyses using biochemical markers or histopathology methods. The upregulation of molecules related to immune modulation, adhesion, or angiogenesis is essential for endometrial receptivity, and progesterone may be involved in this process.^2^ A recently created transcriptomic atlas of the human endometrium at single‐cell resolution clearly demonstrated that the window of implantation (WOI) opens with abrupt and discontinuous transcriptomic activation of the epithelia and stromal fibroblasts.^3^

Endometrial receptivity analysis (ERA, ©Igenomix) is the first commercial test available for clinical analysis of the expression patterns of 248 genes to identify the receptivity status (i.e., receptive or non‐receptive) of the endometrium and determine WOI displacement in a particular patient.^1^, ^4^ Patients with receptive results according to ERA can undergo standard frozen embryo transfer (FET) in a subsequent cycle; in patients with non‐receptive results, adjustment of embryo transfer timing (so‐called personalized embryo transfer, pET) is recommended to improve ART outcomes.^2^, ^5^, ^6^ A recent systematic review and meta‐analysis^7^ demonstrated that one‐third of infertile women had a displaced WOI, and pET may facilitate implantation in patients with recurrent implantation failure caused by non‐receptive endometrium.

There is a growing body of evidence that microbiota, which are specific microbe populations that symbiotically inhabit the host, communicate with the host through humoral signaling molecules, thus influencing overall health status.^8^ Recent studies have suggested connections between microbiota and female reproductive tract disorders, such as bacterial vaginosis, cervical and endometrial cancer, polycystic ovary syndrome, postmenopausal syndrome, endometriosis, endometritis, uterine fibroids, and infertility.^9^, ^10^, ^11^ Throughout the female reproductive tract, *Lactobacillus* spp. are the most frequently identified bacteria. Analyses of the V3–V4–V6 regions in the 16S rRNA gene via next‐generation sequencing (NGS)^12^ revealed that the endometrial microbiome comprises low‐biomass microbiota; moreover, it contains fewer bacteria than the vaginal microbiome.^13^, ^14^ Multiple studies have demonstrated that ART outcomes are affected by altered microbiota in the vagina,^15^, ^16^, ^17^, ^18^, ^19^ uterine cervix,^15^, ^20^, ^21^ and endometrium.^12^, ^22^ The presence of bacterial pathogens associated with chronic endometritis (CE) induces persistent inflammation of the endometrial mucosa, thereby impairing endometrial receptivity through alterations of decidualization and various cellular mechanisms.^23^, ^24^ A recent multicenter prospective study showed that the presence of pathogenic bacteria in the endometrium, together with the depletion of *Lactobacillus*, was associated with implantation failure.^25^

EndomeTRIO (©Igenomix, Valencia, Spain) is a triad of NGS‐based tests including ERA, endometrial microbiome metagenomic analysis (EMMA), and analysis of infectious chronic endometritis (ALICE). EMMA is used to evaluate a patient's endometrial microbiota, and ALICE is performed to identify the pathogenic bacteria associated with CE. The EndomeTRIO has simplified endometrial receptivity testing for clinicians and provides additional information that may help develop a therapeutic strategy for ART. However, the mechanisms responsible for differences in receptive status timing are poorly understood because patient confidentiality considerations have hindered the standardization of algorithms used to predict the WOI with these transcriptomes. Here, we conducted a cross‐sectional study to identify factors that may affect endometrial receptivity array results. We hypothesized that age‐related changes in the endometrial microbiota were involved in the development of endometrial receptivity.

## 2 MATERIALS AND METHODS

### 2.1 Study population

We enrolled infertile patients who were planning to undergo FET at the blastocyst stage and who underwent their first EndomeTRIO test at our clinic during the period from August 2021 to July 2022. All eligible participants had experienced at least 1 unsuccessful embryo transfer (Gardner's grade of 4BB or higher), had at least one remaining frozen blastocyst (Gardner's grade of 4BB or higher), and were given detailed information about the EndomeTRIO test prior to FET. All patients made the decision regarding whether to undergo testing, and informed consent for clinical testing and for inclusion in the study was obtained from all patients. Patients who had used antibiotics, probiotics, or prebiotics within 1 month were excluded from the study.

### 2.2 Endometrial preparation for testing

The EndomeTRIO test was performed in a programmed hormone replacement cycle, in accordance with the routine FET program at our clinic. Endometrial preparation with transdermal estradiol tape (Estrana®, 1.44–2.16 mg every 2 days; Hisamitsu Pharmaceutical Co., Inc.) was started on cycle day 4. A transvaginal ultrasound was performed to assess the endometrium and ovaries after 10–12 days of estradiol. Progesterone was initiated when the endometrium, with a trilaminar appearance, was thicker than 7 mm. To exclude unexpected early progesterone exposure, the administration of progesterone was started within 12 h of the point at which transvaginal ultrasound showed no growing follicles greater than 12 mm in diameter and blood tests confirmed a serum progesterone level <1.0 ng/dL. Blood sample testing was conducted in our laboratory using an Access 2 immunoassay system (Beckman‐Coulter). Exogenous progesterone was administered via the vaginal route using Luteum® vaginal suppositories (400 mg twice per day; ASKA Pharmaceutical Co., Ltd.) in patients who received 2.16 mg of transdermal estradiol or OneCrinone® vaginal gel (90 mg once per day; Merck KGaA) in patients who received 1.44 mg of transdermal estradiol.

### 2.3 Endometrial biopsy

The EndomeTRIO test was performed approximately 108 h after the initial administration of progesterone. After the uterine cervix had been washed with saline solution and the external cervical os had been wiped using a clean cotton ball, an endometrial biopsy was performed using a Pipelle® (Laboratoire CCD). Aspirated tissues (excluding mucus and blood) were placed into a cryotube containing RNAlater (Qiagen), then stored and shipped at 4°C for EndomeTRIO testing, in accordance with the manufacturer's protocol.^1^

### 2.4 Endometrial receptivity analysis

Endometrial tissue was lysed and homogenized with TissueLyser II (Qiagen), and the supernatant was recovered via centrifugation. The patient's RNA was extracted from the supernatant using QIAsymphony SP and QIAsymphony RNA kits (Qiagen), then subjected to reverse transcription to produce cDNA, which was used for library preparation with Ion Torrent (Thermo Fisher Scientific). The expression patterns of 248 genes associated with endometrial receptivity were analyzed using an Ion S5 NGS system (Thermo Fisher Scientific).

### 2.5 Endometrial microbiome metagenomic analysis

A portion of each specimen subjected to ERA was also tested using EMMA and ALICE. Briefly, 25 mg of endometrial tissue were treated with proteinase K at 56°C for 3 h, then separated and lysed with ATL buffer (Qiagen). Subsequently, the tissues were disrupted with a TissueLyser LT (Qiagen) for 5 min at 50 Hz using stainless steel beads. Bacterial nucleic acids from the sample were purified using QIAsymphony (Qiagen), in accordance with the manufacturer's instructions, and quantified with MultiskanGO (Thermo Fisher Scientific).

Hypervariable regions of the gene encoding the bacterial 16S ribosomal subunit (V2–4–8 and V3–6, 7–9) were amplified using an Ion 16S metagenomics kit (Thermo Fisher Scientific). The amplified products were fragmented and barcoded with an Ion Plus Fragment Library kit and Ion Xpress Barcode Adaptors (Thermo Fisher Scientific), in accordance with the manufacturer's instructions. The individual libraries were pooled, and the emulsion polymerase chain reaction was performed using an Ion OneTouch 2 System or an Ion Chef System. Libraries were sequenced with the Ion Torrent S5 XL NGS system (Thermo Fisher Scientific).^12^

### 2.6 Interpretation of the EndomeTRIO test

The ERA results were interpreted as pre‐receptive (profile ≥24 h earlier than the WOI), receptive (profile timed to the WOI), or post‐receptive (profile later than the WOI). The EMMA results were interpreted as pattern 1 (normal microbiome with *Lactobacillus* > 90% and negative for bacterial pathogens), pattern 2 (abnormal microbiome with *Lactobacillus* < 90% and negative for bacterial pathogens causing CE), pattern 3 (abnormal microbiome with *Lactobacillus* < 90% and positive for bacterial pathogens causing CE), pattern 4 (mild dysbiotic microbiome profile), or pattern 5 (microbiome with ultralow biomass). Patterns 2 and 3 differed according to whether bacterial pathogens causing CE were present. The approximate quantity of bacterial DNA was estimated by comparison with the quantity of human DNA per specimen weight, with the requirement that a specific amount of human DNA be present in the specimen. The results were interpreted as pattern 4 when only trace amounts of bacterial DNA were detected; they were interpreted as pattern 5 when there was no amplification of bacterial DNA (equivalent to the negative control containing only water). The results of ALICE were interpreted as positive when bacterial pathogens causing CE were present, as represented by pattern 3 in the EMMA results. Pathogens causing CE included *Escherichia*, *Klebsiella*, *Enterococcus*, *Chlamydia*, *Mycoplasma*, *Ureaplasma*, *Streptococcus*, and *Staphylococcus*.^26^

### 2.7 Statistical analysis

To compare variables across groups, the Student's *t*‐test and analysis of variance were used for normally distributed data. Comparisons of categorical variables were performed in contingency table analysis using Pearson's χ^2^ test. Single and multivariable logistic regression analyses were used to determine associations between the ERA results and patient age, body mass index (BMI), and EMMA results. The inclusion of variables in the analyses was based on existing knowledge concerning risk factors for WOI displacement. Odds ratios and 95% confidence intervals related to each variable were calculated. The Wald test was used to assess the overall association. All statistical analyses were conducted using JMP software v. 15.2.1 (SAS Institute Inc.). All tests were two‐tailed, and *p* < 0.05 was considered statistically significant.

## 3 RESULTS

**FIGURE 1:**
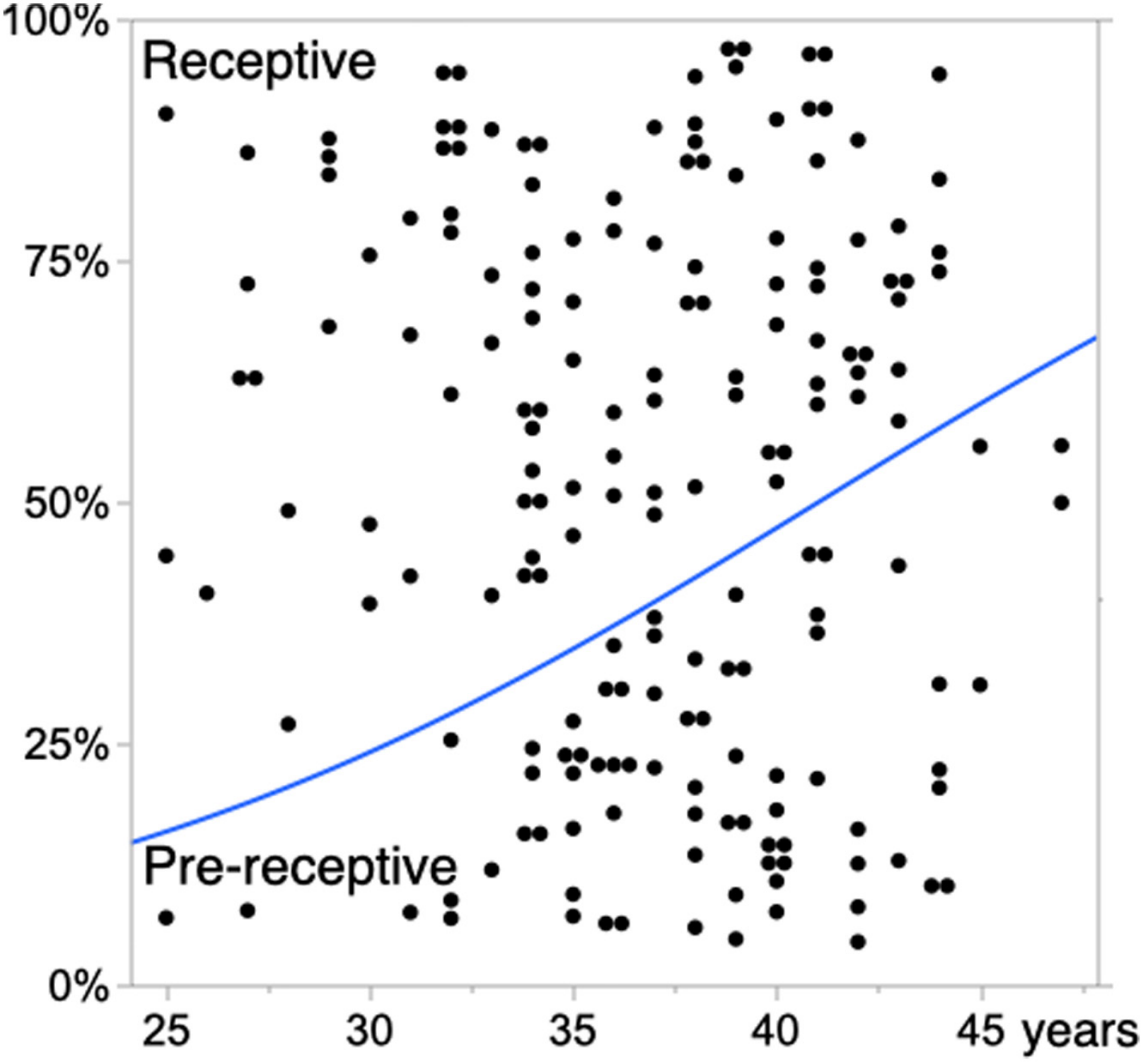
Age‐related increase in pre‐receptive endometrium and decrease in receptive endometrium, interpreted using endometrial receptivity analysis (ERA). The horizontal axis represents patient age, and the vertical axis represents the proportion of endometrial receptivity interpreted using ERA. *p* = 0.0018 in logistic regression analysis with the Wald test.

**FIGURE 2:**
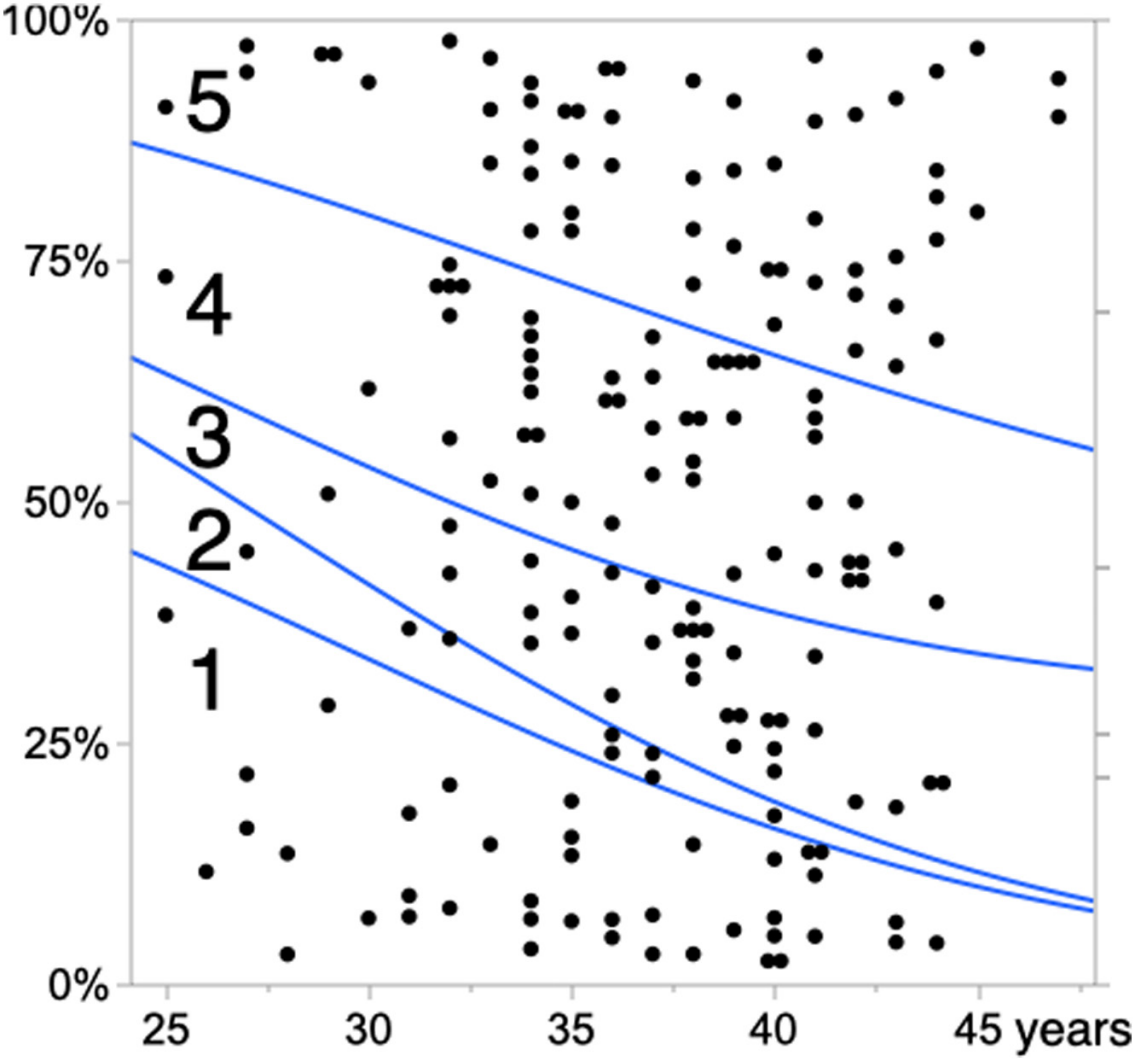
Age‐related increase in endometrium with ultralow biomass microbiome and decrease in endometrium with *Lactobacillus*‐dominant microbiota, interpreted using endometrial microbiome metagenomic analysis (EMMA). The horizontal axis represents patient age, and the vertical axis represents the proportions of endometrial microbiota patterns interpreted using EMMA. Numbers 1–5 in the graph represent patterns 1–5. *p* = 0.0317 in logistic regression analysis with the Wald test.

In this study, we included a total of 185 EndomeTRIO tests for 185 eligible patients aged 25 to 47 years. The results of ERA were receptive in 111 patients (the Receptive ERA group) and pre‐receptive in 74 patients (the Pre‐receptive ERA group); no patients had post‐receptive ERA results. The results of the EMMA test showed pattern 1 in 40 patients, pattern 2 in eight patients, pattern 3 in 32 patients, pattern 4 in 49 patients, and pattern 5 in 56 patients. In a comparison between the Receptive ERA and Pre‐receptive ERA groups, BMI and serum progesterone levels at initial progesterone administration showed no significant differences. However, patient age was significantly greater in the Pre‐receptive ERA group, and the distribution of the EMMA results was significantly different; the Pre‐receptive ERA group showed a decrease in pattern 1 and an increase in pattern 5 (Table 1). Age was associated with a significant decrease in receptivity according to ERA results and a significant decrease in pattern 1 according to EMMA (Figure 1). The EMMA results were significantly associated with the ERA results. Patients with pattern 1 had a higher rate of receptive ERA results and a lower rate of pre‐receptive ERA results; these rates were reversed in patients with pattern 5 (Figure 2). Multivariable logistic regression analysis revealed that patient age and EMMA results were both significant independent predictive factors for pre‐receptive ERA results (Table 2).

**TABLE 1:** Patient characteristics and EMMA results in the receptive ERA and pre‐receptive ERA groups.

ERA results	Total	(*n* = 185)	Receptive	(*n* = 111)	Pre‐receptive	(*n* = 74)	*p*
	M ± SD		M ± SE	[95% CI]	M ± SE	[95% CI]	
Age (years)	36.9 ± 4.7		36.0 ± 0.5	[35.2–36.9]	38.2 ± 0.5	[37.1–39.2]	0.0021
BMI (kg/m^2^)	21.8 ± 3.0		21.6 ± 0.4	[21.0–22.1]	22.1 ± 0.3	[21.4–22.7]	0.2847
No. of previous ET	2.3 ± 1.9		2.3 ± 0.2	[1.9–2.6]	2.4 ± 0.2	[2.0–2.8]	0.6061
Serum P levels (ng/mL)	0.26 ± 0.20		0.26 ± 0.02	[0.22–0.30]	0.26 ± 0.02	[0.21–0.31]	0.9964
EMMA results							
Pattern 1	40	(21.6%)	31	(27.9%)	9	(12.2%)	0.0074
Pattern 2	8	(4.3%)	7	(6.3%)	1	(1.4%)	
Pattern 3	32	(17.3%)	17	(15.3%)	15	(20.3%)	
Pattern 4	49	(26.5%)	31	(27.9%)	18	(24.3%)	
Pattern 5	56	(30.3%)	25	(22.5%)	31	(41.9%)	

*Note*: Continuous variables (patient age, BMI, serum *p* levels) were compared using Student's *t*‐test and analysis of variance; categorical data (EMMA results) were compared using Pearson's chi‐square test.

Abbreviations: BMI, body mass index; CI, confidence interval; CI, confidence interval; EMMA, endometrial microbiome metagenomic analysis; ERA, endometrial receptivity analysis; ET, embryo transfer; M, mean; P, progesterone; SD, standard deviation; SE, standard error of the mean.

**TABLE 2:** Factors associated with probable pre‐receptive ERA results.

	Crude	[95% CI]	*p*	Adjusted	[95% CI]	*p*
	OR			OR		
Patient age (years)	1.11	[1.03–1.19]	0.0018	1.08	[1.01–1.16]	0.0351
BMI (kg/m^2^)	1.06	[0.96–1.16]	0.2834	1.06	[0.95–1.18]	0.3570
EMMA results			0.0115			0.0376
Pattern 1	1	[Reference]		1	[Reference]	
Pattern 2	0.49	[0.05–4.54]	0.5044	0.50	[0.05–4.77]	0.5232
Pattern 3	3.04	[1.10–8.40]	0.0290	2.62	[0.92–7.38]	0.0656
Pattern 4	2.00	[0.78–5.13]	0.1429	1.86	[0.71–4.87]	0.2016
Pattern 5	4.27	[1.72–10.61]	0.0010	3.82	[1.49–9.82]	0.0039

*Note*: Crude data based on single logistic regression analyses; adjusted data based on multivariable logistic regression analysis. Significance of parameters tested using the *p*‐values of respective Wald tests. The values of Patterns 2–5 in the EMMA results were based on Pattern 1 as a reference.

Abbreviations: BMI, body mass index; CI, confidence interval; EMMA, endometrial microbiome metagenomic analysis; ERA, endometrial receptivity analysis; OR, odds ratio.

## 4 DISCUSSION

In this observational study, the incidence of pre‐receptive endometrium significantly increased with age. Pre‐receptive endometrium was also significantly associated with a decreased proportion of *Lactobacillus*‐dominant microbiota and with the presence of dysbiotic microbiota in the endometrium. In particular, women who had a microbiome with ultralow biomass were likely to have pre‐receptive endometrium. Both aging and dysbiotic microbiota were independent predictive factors for WOI displacement. After adjustment for other factors, pattern 5 in the EMMA test was most strongly associated with the odds of pre‐receptive ERA results. We estimated that the odds of pre‐receptive ERA results were 3.8‐fold higher for patients with pattern 5 on EMMA than for patients with pattern 1 on EMMA.

### 4.1 The timing of endometrial biopsy

ERA must be conducted with caution in a strictly standardized setting because the results can be affected by various factors. The recommended timing of an endometrial biopsy for ERA testing is 120 h after progesterone administration.^27^ However, the timing of endometrial biopsy can slightly vary from 120 h after initial progesterone administration; it is usually performed after 5 days (P + 5). Although an earlier or later biopsy decreases the proportion of patients with receptive ERA results, the diagnostic algorithm for ERA can predict the WOI with high accuracy.^26^ Although endometrial biopsy 108 h after initial progesterone administration did not deviate from recommendations, we guess that biopsy collection 12 h earlier may help expose the effects of age and endometrial microbiota on endometrial receptivity, leading to an increase in the proportion of patients with pre‐receptive ERA results. Although the timing of the biopsy is important, there is a greater need to confirm that no endogenous progesterone exposure occurred immediately before the administration of exogenous progesterone; activation of progesterone receptors can trigger the initial development of endometrial receptivity.^2^ Endometrial receptivity is mainly regulated by ovarian steroids throughout the cycle. A recent systematic review and meta‐analysis did not reveal a significant change in the rate of pregnancy after in vitro fertilization cycles using ERA.^28^ However, all studies included in that meta‐analysis were retrospective and did not measure endogenous progesterone in ERA cycles; half of the studies included natural cycle FETs. It is difficult to accurately determine the timing of progesterone secretion in the natural cycle solely by using urinary luteinizing hormone tests.^29^ We believe that ERA and subsequent FETs to evaluate the efficacy of ERA should be performed in hormone replacement cycles.

### 4.2 Age‐related changes of endometrial receptivity

The timing of WOI is unique to each individual and can persist for up to 40 months after it has been determined.^4^ Although the mechanism of age‐related changes in endometrial receptivity is unclear,^30^ a recent endometrial transcriptomic data analysis revealed that age‐related changes begin at the age of 35 years; these changes include upregulation of genes involved in ciliary processes as well as growth factor dysregulation.^31^ Our results are consistent with an age‐related delay in the development of receptive endometrium. Although obesity is reportedly associated with significant endometrial transcriptomic differences relative to non‐obesity,^32^ the effect of increased BMI was not apparent in our study, probably because participants were limited to Japanese women, among whom obesity is less common.^33^

### 4.3 Microbiome‐related changes of endometrial receptivity

The use of the EndomeTRIO test for simultaneous assessment of endometrial receptivity and endometrial microbiota is less invasive and less costly for patients. We found that microbiota with ultralow biomass (EMMA pattern 5) have detrimental effects on endometrial receptivity. According to a recent systematic review,^34^ vaginal, cervical, and endometrial dysbiosis, which is characterized by decreases in core commensal bacteria along with increases in opportunistic and pathogenic microbes, may have negative effects on ART outcomes. However, past relevant studies have contained inconsistencies and missing information. Factors affecting reproductive outcomes include the diversity of bacterial species, the predominance of *Lactobacillus*, the proportions of various *Lactobacillus* species, and the presence of specific other bacteria. Cela et al.^10^ demonstrated that dysbiotic endometrial tissue had higher levels of inflammatory molecules and lower levels of anti‐inflammatory or growth factors (e.g., interleukin 10 and insulin‐like growth factor 1); both changes were inversely correlated with the proportion of *Lactobacillus*. Because these anti‐inflammatory and growth factors are involved in the development of receptive endometrium, *Lactobacillus*‐dominant microbiota very likely participate in the process of achieving endometrial receptivity. In the present study, the crude odds ratio of pattern 3 (positive for pathogens causing CE) for pre‐receptive ERA results was statistically significant (*p* = 0.0290), but the adjusted odds ratio was not (*p* = 0.0656). Although CE caused by pathogenic bacteria can impair the process of achieving endometrial receptivity, such inflammation may be one of multiple causes of pre‐receptive endometrium. The crucial difference between patterns 2–4 and pattern 5 is the presence of bacterial flora. Patterns 2–4 have detectable microbiota, albeit with less *Lactobacillus*, whereas pattern 5 almost completely lacks microbiota, including *Lactobacillus*. Considering our findings that EMMA patterns 2–4 showed weaker associations with the results of ERA, we suspect that the quantity (rather than the proportion) of *Lactobacillus* affects endometrial receptivity.

There has been considerable attention toward the impact of the gut microbiome on a variety of diseases.^35^ The gut microbiota can maintain intestinal epithelial homeostasis and promote health. The surface macromolecules or metabolites of microbes, including *Lactobacillus*, interact with host cells to produce various cytokines and chemokines that alleviate inflammation and enhance epithelial function. The bacterial composition within each organ system is influenced by individual factors including age,^36^ genetics, ethnicity, diet, probiotics, prebiotics, lifestyle, and exposure to antibiotics or other chemicals.^37^ Nevertheless, the roles of endometrial microbiota in reproduction and the roles of probiotics in infertility management have not been extensively studied. The endometrial microbiota varies among individuals. Aging is reportedly accompanied by a decrease in the alpha diversity of the endometrial microbiome.^38^ The phase of the menstrual cycle may also have a slight influence on the endometrial microbiota; a difference between the follicular and luteal phases has been reported^38^; a significant decrease in diversity and evenness, occurring around ovulation and continuing into the secretory phase, has also been reported.^39^ Moreover, the endometrial microbiota may be altered by exogenous hormones, such as the hormones used in ovarian stimulation, progesterone supplementation,^14^ or various types of ovulation induction and luteal support.^40^, ^41^

### 4.4 Alternative strategy to evaluate endometrial receptivity

Because the proportion of infertility patients with EMMA pattern 1 is approximately 25% in ordinary clinical settings, more than half of patients will require therapeutic intervention (e.g., probiotics, prebiotics, or antibiotics). Antibiotics used for the treatment of CE may induce transient elimination of pathogens as well as *Lactobacillus* spp., thus resulting in a microbiome with ultralow biomass; this type of microbiome is often observed in re‐examination of EMMA results, which is recommended for patients with positive results concerning pathogenic bacteria. Nevertheless, it remains unknown whether ERA results would be consistent after treatment to reconstruct the ideal endometrial microbiome. Therefore, careful follow‐up and re‐examination may be indispensable, particularly for patients with pre‐receptive ERA results accompanied by abnormal EMMA results. Considering our findings, we propose an alternative strategy to evaluate endometrial receptivity in ART, namely, conducting EMMA prior to ERA. For patients with abnormal endometrial microbiota and positive results concerning bacterial pathogens, ERA should be performed after pathogen elimination has been confirmed using appropriate antibiotics and subsequent probiotics. For patients with a normal or dysbiotic microbiome and no bacterial pathogens, ERA can be performed in the following cycle, with probiotics administered if necessary. Although repeated biopsies would slightly increase the patient burden, this strategy may help to maximize the likelihood of performing FET at the appropriate WOI.

### 4.5 Conclusions

In this study, we found that aging was accompanied by a decrease in *Lactobacillus*‐dominant microbiota; both aging and endometrial microbiota with ultralow biomass were significantly associated with pre‐receptive endometrium. These findings suggest that the quantity (rather than the proportion) of *Lactobacillus* in the endometrium is involved in the development of endometrial receptivity. A limitation of the present study is that it constituted a small‐scale cross‐sectional study at a single private clinic. Nevertheless, these findings are robust because the study was conducted in a strictly standardized setting where endogenous progesterone exposure was excluded. Our findings can assist clinicians in analyses of endometrial receptivity and endometrial microbiota, which may improve therapeutic outcomes in ART. Additional prospective cohort studies are necessary to evaluate the effects of probiotics on endometrial receptivity array results (based on endometrial microbiota composition) and reproductive outcomes. Moreover, extensive research is needed to expand the current understanding of molecular and pathophysiological interactions between microbiota, particularly *Lactobacillus*, and endometrial receptivity.

## CONFLICT OF INTEREST STATEMENT

Human rights statements and informed consent: All procedures were performed in accordance with the ethical standards of the responsible committee on human experimentation *(institutional and national)* and with the Helsinki Declaration of 1964 and its later amendments. Informed consent was obtained from all participants *included in the study*. The study protocol was approved by the Ethics Committee of our clinic. The authors declare no conflicts of interest for this article. We certify that no person other than the authors has made substantial contributions to the work.

## References

[1] Diaz‐Gimeno P , Horcajadas JA , Martinez‐Conejero JA , Esteban FJ , Alama P , Pellicer A , et al. A genomic diagnostic tool for human endometrial receptivity based on the transcriptomic signature. Fertil Steril. 2011;95(1):50–60. e1–15. 20619403 10.1016/j.fertnstert.2010.04.063

[2] Ruiz‐Alonso M , Blesa D , Diaz‐Gimeno P , Gomez E , Fernandez‐Sanchez M , Carranza F , et al. The endometrial receptivity array for diagnosis and personalized embryo transfer as a treatment for patients with repeated implantation failure. Fertil Steril. 2013;100(3):818–24. 23756099 10.1016/j.fertnstert.2013.05.004

[3] Wang W , Vilella F , Alama P , Moreno I , Mignardi M , Isakova A , et al. Single‐cell transcriptomic atlas of the human endometrium during the menstrual cycle. Nat Med. 2020;26(10):1644–53. 32929266 10.1038/s41591-020-1040-z

[4] Diaz‐Gimeno P , Ruiz‐Alonso M , Blesa D , Bosch N , Martinez‐Conejero JA , Alama P , et al. The accuracy and reproducibility of the endometrial receptivity array is superior to histology as a diagnostic method for endometrial receptivity. Fertil Steril. 2013;99(2):508–17. 23102856 10.1016/j.fertnstert.2012.09.046

[5] Hashimoto T , Koizumi M , Doshida M , Toya M , Sagara E , Oka N , et al. Efficacy of the endometrial receptivity array for repeated implantation failure in Japan: a retrospective, two‐centers study. Reprod Med Biol. 2017;16(3):290–6. 29259480 10.1002/rmb2.12041 PMC5715887

[6] Jia Y , Sha Y , Qiu Z , Guo Y , Tan A , Huang Y , et al. Comparison of the effectiveness of endometrial receptivity analysis (ERA) to guide personalized embryo transfer with conventional frozen embryo transfer in 281 Chinese women with recurrent implantation failure. Med Sci Monit. 2022;22(28):e935634. 10.12659/MSM.935634 PMC8957643 35314667

[7] Liu Z , Liu X , Wang M , Zhao H , He S , Lai S , et al. The clinical efficacy of personalized embryo transfer guided by the endometrial receptivity array/analysis on IVF/ICSI outcomes: a systematic review and meta‐analysis. Front Physiol. 2022;27(13):841437. 10.3389/fphys.2022.841437 PMC9092494 35574479

[8] Ciebiera M , Esfandyari S , Siblini H , Prince L , Elkafas H , Wojtyła C , et al. Nutrition in gynecological diseases: current perspectives. Nutrients. 2021;13(4):1178. 33918317 10.3390/nu13041178 PMC8065992

[9] Elkafas H , Wall M , Al‐Hendy A , Ismail N . Gut and genital tract microbiomes: dysbiosis and link to gynecological disorders. Front Cell Infect Microbiol. 2022;16(12):1059825. 10.3389/fcimb.2022.1059825 PMC9800796 36590579

[10] Cela V , Daniele S , Obino MER , Ruggiero M , Zappelli E , Ceccarelli L , et al. Endometrial dysbiosis is related to inflammatory factors in women with repeated implantation failure: a pilot study. J Clin Med. 2022;11(9):2481. 35566605 10.3390/jcm11092481 PMC9101226

[11] Rowe M , Veerus L , Trosvik P , Buckling A , Pizzari T . The reproductive microbiome: an emerging driver of sexual selection, sexual conflict, mating systems, and reproductive isolation. Trends Ecol Evol. 2020;35(3):220–34. 31952837 10.1016/j.tree.2019.11.004

[12] Moreno I , Codoner FM , Vilella F , Valbuena D , Martinez‐Blanch JF , Jimenez‐Almazan J , et al. Evidence that the endometrial microbiota has an effect on implantation success or failure. Am J Obstet Gynecol. 2016;215(6):684–703. 27717732 10.1016/j.ajog.2016.09.075

[13] Chen C , Song X , Wei W , Zhong H , Dai J , Lan Z , et al. The microbiota continuum along the female reproductive tract and its relation to uterine‐related diseases. Nat Commun. 2017;8(1):875. 29042534 10.1038/s41467-017-00901-0 PMC5645390

[14] Carosso A , Revelli A , Gennarelli G , Canosa S , Cosma S , Borella F , et al. Controlled ovarian stimulation and progesterone supplementation affect vaginal and endometrial microbiota in IVF cycles: a pilot study. J Assist Reprod Genet. 2020;37(9):2315–26. 32671734 10.1007/s10815-020-01878-4 PMC7492325

[15] Graspeuntner S , Bohlmann MK , Gillmann K , Speer R , Kuenzel S , Mark H , et al. Microbiota‐based analysis reveals specific bacterial traits and a novel strategy for the diagnosis of infectious infertility. PLoS One. 2018;13(1):e0191047. 29315330 10.1371/journal.pone.0191047 PMC5760088

[16] Kyono K , Hashimoto T , Nagai Y , Sakuraba Y . Analysis of endometrial microbiota by 16S ribosomal RNA gene sequencing among infertile patients: a single‐center pilot study. Reprod Med Biol. 2018;17(3):297–306. 30013432 10.1002/rmb2.12105 PMC6046523

[17] Koedooder R , Mackens S , Budding A , Fares D , Blockeel C , Laven J , et al. Identification and evaluation of the microbiome in the female and male reproductive tracts. Hum Reprod Update. 2019;25(3):298–325. 30938752 10.1093/humupd/dmy048

[18] Bernabeu A , Lledo B , Díaz MC , Lozano FM , Ruiz V , Fuentes A , et al. Effect of the vaginal microbiome on the pregnancy rate in women receiving assisted reproductive treatment. J Assist Reprod Genet. 2019;36(10):2111–9. 31446545 10.1007/s10815-019-01564-0 PMC6823330

[19] Karaer A , Doğan B , Günal S , Tuncay G , Arda Düz S , Ünver T , et al. The vaginal microbiota composition of women undergoing assisted reproduction: a prospective cohort study. BJOG. 2021;128(13):2101–9. 34053157 10.1111/1471-0528.16782

[20] Hao X , Li P , Wu S , Tan J . Association of the cervical microbiota with pregnancy outcome in a subfertile population undergoing in vitro fertilization: a case‐control study. Front Cell Infect Microbiol. 2021;23(11):654202. 10.3389/fcimb.2021.654202 PMC8495128 34631595

[21] Villani A , Fontana A , Barone S , de Stefani S , Primiterra M , Copetti M , et al. Identifying predictive bacterial markers from cervical swab microbiota on pregnancy outcome in woman undergoing assisted reproductive technologies. J Clin Med. 2022;11(3):680. 35160131 10.3390/jcm11030680 PMC8836651

[22] Kyono K , Hashimoto T , Kikuchi S , Nagai Y , Sakuraba Y . A pilot study and case reports on endometrial microbiota and pregnancy outcome: an analysis using 16S rRNA gene sequencing among IVF patients, and trial therapeutic intervention for dysbiotic endometrium. Reprod Med Biol. 2018;18(1):72–82. 30655724 10.1002/rmb2.12250 PMC6332758

[23] Kitaya K , Yasuo T . Aberrant expression of selectin E, CXCL1, and CXCL13 in chronic endometritis. Mod Pathol. 2010;23(8):1136–46. 20495539 10.1038/modpathol.2010.98

[24] Di Pietro C , Cicinelli E , Guglielmino MR , Ragusa M , Farina M , Palumbo MA , et al. Altered transcriptional regulation of cytokines, growth factors, and apoptotic proteins in the endometrium of infertile women with chronic endometritis. Am J Reprod Immunol. 2013;69(5):509–17. 23351011 10.1111/aji.12076

[25] Moreno I , Garcia‐Grau I , Perez‐Villaroya D , Gonzalez‐Monfort M , Bahçeci M , Barrionuevo MJ , et al. Endometrial microbiota composition is associated with reproductive outcome in infertile patients. Microbiome. 2022;10(1):1. 34980280 10.1186/s40168-021-01184-w PMC8725275

[26] Moreno I , Cicinelli E , Garcia‐Grau I , Gonzalez‐Monfort M , Bau D , Vilella F , et al. The diagnosis of chronic endometritis in infertile asymptomatic women: a comparative study of histology, microbial cultures, hysteroscopy, and molecular microbiology. Am J Obstet Gynecol. 2018;218(6):602 e1–e16. 10.1016/j.ajog.2018.02.012 29477653

[27] Stankewicz T , Valbuena D , Ruiz‐Alonso M . Inter‐cycle consistency versus test compliance in endometrial receptivity analysis test. J Assist Reprod Genet. 2018;35(7):1307–8. 29804174 10.1007/s10815-018-1212-7 PMC6063832

[28] Arian SE , Hessami K , Khatibi A , To AK , Shamshirsaz AA , Gibbons W . Endometrial receptivity array before frozen embryo transfer cycles: a systematic review and meta‐analysis. Fertil Steril. 2023;119(2):229–38. 36414088 10.1016/j.fertnstert.2022.11.012

[29] Dozortsev DI , Diamond MP . Luteinizing hormone‐independent rise of progesterone as the physiological trigger of the ovulatory gonadotropins surge in the human. Fertil Steril. 2020;114(2):191–9. 32741458 10.1016/j.fertnstert.2020.06.016

[30] Fogle RH , Li A , Paulson RJ . Modulation of HOXA10 and other markers of endometrial receptivity by age and human chorionic gonadotropin in an endometrial explant model. Fertil Steril. 2010;93(4):1255–9. 19131054 10.1016/j.fertnstert.2008.11.002

[31] Devesa‐Peiro A , Sebastian‐Leon P , Parraga‐Leo A , Pellicer A , Diaz‐Gimeno P . Breaking the ageing paradigm in endometrium: endometrial gene expression related to cilia and ageing hallmarks in women over 35 years. Hum Reprod. 2022;37(4):762–76. 35085395 10.1093/humrep/deac010

[32] Comstock IA , Diaz‐Gimeno P , Cabanillas S , Bellver J , Sebastian‐Leon P , Shah M , et al. Does an increased body mass index affect endometrial gene expression patterns in infertile patients? A functional genomics analysis. Fertil Steril. 2017;107(3):740–8 e2. 27919438 10.1016/j.fertnstert.2016.11.009

[33] Saito I . Epidemiological evidence of type 2 diabetes mellitus, metabolic syndrome, and cardiovascular disease in Japan. Circ J. 2012;76(5):1066–73. 22453006 10.1253/circj.cj-11-1519

[34] Dube R , Kar SS . Genital microbiota and outcome of assisted reproductive treatment–a systematic review. Life (Basel). 2022;12(11):1867. 36431002 10.3390/life12111867 PMC9693990

[35] Conway J , Duggal NA . Ageing of the gut microbiome: potential influences on immune senescence and inflammageing. Ageing Res Rev. 2021;68:101323. 33771720 10.1016/j.arr.2021.101323

[36] Odamaki T , Kato K , Sugahara H , Hashikura N , Takahashi S , Xiao JZ , et al. Age‐related changes in gut microbiota composition from newborn to centenarian: a cross‐sectional study. BMC Microbiol. 2016;25(16):90. 10.1186/s12866-016-0708-5 PMC4879732 27220822

[37] Khan S , Luck H , Winer S , Winer DA . Emerging concepts in intestinal immune control of obesity‐related metabolic disease. Nat Commun. 2021;12(1):2598. 33972511 10.1038/s41467-021-22727-7 PMC8110751

[38] Wang R , Zhou G , Wu L , Huang X , Li Y , Luo B , et al. The microbial composition of lower genital tract may affect the outcome of in vitro fertilization‐embryo transfer. Front Microbiol. 2021;1(12):729744. 10.3389/fmicb.2021.729744 PMC8518997 34659157

[39] Odawara K , Akino R , Sekizawa A , Sakamoto M , Yuriko S , Tanaka K , et al. Examination of clinical factors affecting intrauterine microbiota. Reprod Fertil. 2021;2(1):1–6. 35128428 10.1530/RAF-20-0030 PMC8812463

[40] Vomstein K , Reider S , Böttcher B , Watschinger C , Kyvelidou C , Tilg H , et al. Uterine microbiota plasticity during the menstrual cycle: differences between healthy controls and patients with recurrent miscarriage or implantation failure. J Reprod Immunol. 2022;151:103634. 35550495 10.1016/j.jri.2022.103634

[41] Bermejo A , Cerrillo M , Ruiz‐Alonso M , Blesa D , Simon C , Pellicer A , et al. Impact of final oocyte maturation using gonadotropin‐releasing hormone agonist triggering and different luteal support protocols on endometrial gene expression. Fertil Steril. 2014;101(1):138–46. e3. 24182413 10.1016/j.fertnstert.2013.09.033

